# Health-Resorts in the West of England and South Wales. Cheltenham

**Published:** 1893-12

**Authors:** J. H. Garrett

**Affiliations:** Medical Officer of Health, Cheltenham


### Health-Resorts in the West of England and South Wales.

### IX.

### CHELTENHAM.

BY

J. H. Garrett, M.D., B.S. Dur., D.P.H. Camb.,
*Medical Officer of Health, Cheltenham.*

The Manor and Hundred of Cheltenham is of very ancient foundation. In Domesday Book we learn that "King Edward (the Confessor) held Cheltenham. There are eight hydes and a-half: Reinbald holds one and a-half, which belongs to the church. There are three plough tillages in demesne, and twenty villeins and ten bordars, and seven servi and eighteen plough tillages. In the reign of King Edward it paid £9 5s. and three thousand loaves for the King's dogs. It now pays £20, twenty cows, twenty hogs, and 16/- in lieu of bread for the dogs."

Thus it is evident that at the time of the Norman Conquest, and previously, Cheltenham was a place of importance. It has, however, suffered many vicissitudes and changes. Previous to the Reformation, Leland describes it as a large town, having a market and belonging to the King. In the time of Charles I. it was defended as Royal property, and is stated to have then had a population of 1,500: but being taken by the Roundhead party, it was evacuated by the Royalist residents, and so dwindled down to an insignificant village; the number of births and deaths annually placed upon the parish register for many years after this time prove the population to have been very small, probably it did not exceed 300 or 400.

Attention was first called to a certain saline spring at Cheltenham by flocks of pigeons, which were noticed to settle about the spring, being attracted thither by the deposit of salts upon the earth at the water's edge. Some of the inhabitants afterwards took doses of the water, which was found to be aperient; and in 1738 an enterprising resident enclosed the "Old Well," and began to charge a fee for the water, whilst at the same time he planted some avenues of trees and so laid the foundation of the later Spa. From this period visitors began to arrive at Cheltenham; the fame of its waters rapidly spread abroad, and the fortune of the place was made when King George the Third, by the advice of his physician, Sir George Baker, came in 1788 to drink the waters at Cheltenham. During his stay his Majesty's health was greatly improved, the tide of favour and fashion set strongly towards the place, which grew with amazing rapidity and entered at once into a very successful rivalry with Bath. All the social exercises and amenities of the latter place were put into practice here, and a Master of Ceremonies was appointed at a high salary. Spa buildings arose in numerous localities, where fresh wells were sunk for collection of the water, as the quantity available at the original spring was found quite inadequate to the demand; in fact, for a short time the whole of the available water was drunk up by eleven o'clock in the morning, a crowd of eager patients besieging the pump room, each one jostling his neighbour in fear of being unable to obtain his matutinal dose. Baths were also started, and crystalline salts were prepared by evaporation of the water. Building now went on very rapidly, the population went up by leaps and bounds, and the beautiful villa town as it at present stands was the ultimate result.

After a long run of a very lively success, we at length find the fortunes of Cheltenham and all similar watering places decidedly on the wane. Water drinking for all and sundry complaints was going out of vogue, and the throngs of gay visitors that for so many seasons had with the swallows been wont to appear in Cheltenham ceased to arrive, and the spas or pump rooms were one after another closed, pulled down, left to decay, or converted to some other use; and at the present moment the natural mineral waters can only be obtained at the Montpellier and Pittville Spas, and at the proprietary establishment of Cambray Spa. It is stated that at one time as many as 100 wells were in use, though some of these were connected together with pipes and were to all intents and purposes merely collecting reservoirs. The mineral water interest has become dormant, notwithstanding that it was this interest alone which called this fine town into existence; so dormant, indeed, has it lately been, that the main reminder of the fact of Cheltenham having ever been famous for its medicinal waters is the pigeon—the first discoverer of the virtues of the waters—which sits with great constancy where it has been placed as crest in the Borough Arms.

There are many places with mineral waters that have not the natural advantages of Cheltenham as a healthful and pleasant place of residence, and there are very few towns in England laying claim to the title of health-resort that have so much to boast of in this respect. A study of the geology of the district shows the town to stand partly upon the bare clay of the upper lias and partly upon the sandy drift by which the clay is in places covered. Thus it is easy to find a house built either upon the warmer sandy subsoil, or upon the colder but less pervious clay; and according to the dictates of his personal experience, the house-seeker will choose the site that is likely best to suit him. Within a couple of miles of the centre of the town, on the east and south sides, the lias formation ends, and the oolitic limestone rocks of the Cotswold hills rise into a bold escarpment. The outliers called Chosenhill or Churchdown to the south, and Bredon Hill and Oxenton Hill to the north, help, with the more or less straight ranging line of the Cotswolds proper, to form a wide semicircle of hills on the one side of the town, while the whole western half of the circle is open to the Severn valley. Thus, whilst the town is somewhat sheltered from the colder winds, it is by no means shut in by hills, but lies well exposed to the sunlight and the breeze. The name Cheltenham denotes a town upon rising ground, and from the suburb of Charlton Kings on the extreme east there is in fact a gradual fall to the west—that is, towards the river Severn, which lies seven miles away. Drainage is consequently rendered easy, and is further facilitated by the existence of the stream called the Chelt, which passes through the town in the direction above indicated, to join the Severn about half-way between Tewkesbury and Gloucester. The actual elevation of the town above mean sea-level varies from about 180 feet in its lowest parts to 250 in its higher parts, whilst the hill called Battledown on the north-east border of the borough rises to upwards of 500 feet. Battledown is covered with a sprinkling of villas, and villas have also been built upon some of the higher hills behind, as at Leckhampton and Cleeve hills, and it is possible to reside in the immediate neighbourhood of Cheltenham at elevations varying to upwards of 1,000 feet, the highest point of the Cotswolds being attained on Cleeve Cloud at 1,080 feet, four miles from the centre of Cheltenham. The building of a good hotel at this higher altitude has lately been considered, and other villas will doubtless be built in these "regions of upper air" when a demand for them arises.

The views obtainable from the hills around are hardly to be equalled in extent in any other place in England. A magnificent panorama is presented, embracing the broad Severn valley, in which one can see the towers of Gloucester, Tewkesbury, and Worcester, the valley being bounded on its opposite side by remote hills. Malvern is distinctly visible at a distance of 20 miles, and the fine mountain line which takes its name from that town is almost always visible, extending southward and being continued by the hills of the Forest of Dean. Beyond these again, the Welsh mountains in the counties of Brecknock and Radnor are visible on a clear day; and from the higher points of the Cotswolds the top of the Sugar Loaf

Mountain, sixty miles to the westward, crops up in the distant background with great distinctness. The view to the north only finds its limits in the Lickey and Clent hills at and beyond Bromsgrove, and the Clee hills in far-away Shropshire. From the top of Battledown, or from Leckhampton Hill, the whole of Cheltenham lies in sight. In proportion to the number of its inhabitants the town occupies an immense area, consisting in large part of detached villas with gardens and trees, which fact has earned for it the *sobriquet* of "The Garden Town of England." Its houses are in the main of stone or light-painted stucco; and owing to the absence of factory smoke, the whole place presents a wonderfully clean and *piquant* appearance, with its group of pointed spires in the centre and its white terraces, crescents, and squares. Coming now into the town itself, we find many broad thoroughfares, with wide foot-pavements, most of them being planted with trees like the boulevards of a French city. The most notable street in Cheltenham is perhaps the Promenade, where are double rows of large trees, shading a row of splendid shops, beyond which is the New Club and the glass-covered building of the Winter Gardens on the one side, and on the other side a strip of pretty public gardens, containing a beautiful fountain, lying in front of a terrace of tall dwelling-houses. The road rises gradually to the Queen's Hotel, which, with two cannons posted in front of it, dominates the long avenues and wide pavements of the street. The High Street is an extension of the first original old street of Cheltenham, being the high road from London. It is about two miles long, and is flanked with numerous shops, which afford a cheap market for all kinds of goods. The poor neighbourhood is chiefly off the bottom end of the High Street, and lying nearly all together, forms a quarter separate and distinct from the main town. The necessity for large open spaces is not keenly felt, nevertheless there exist two public parks; namely, the Agg-Gardner Recreation Ground and the Naunton Park, the latter recently opened, and both devoted more particularly to the pleasures of the poorer part of the community. Besides these, there are the Montpellier Gardens and the Pittville Gardens, both the property of the Corporation.

In the former, where in recent years the ground has been chiefly devoted to tennis and archery clubs, it is proposed that the new baths and spa buildings shall be placed. The beautifully planted Pittville Gardens have recently been greatly extended by the purchase and laying-out of an adjoining estate; here a new lake, a third of a mile long, has been also made; it is available for boating and provides safe skating in time of frost.

Cheltenham is well supplied with places of entertainment. It has a new Theatre and Opera House, which is said to be as conveniently constructed as any theatre outside the Metropolis, and here are performed in a constant round all the best productions of the London stage. The Winter Garden, the Assembly Rooms, and the Rotunda, put here in their order of size, offer accommodation for concerts, lectures, large receptions, and other entertainments; whilst the long series of balls held in the Assembly Rooms through the winter season have given a notoriety to the town. Cheltenham is also the centre of a hunting district. The Cotswold hounds have their kennels here, and meets take place in the immediate vicinity several times a week during the season. On the top of Cleeve Hill is a capital set of golf links, kept up by the golf club. The bracing effect of the scramble round these links, which measure three and a-half miles from first tee off to last hole, is very apparent, and every year sees an increase in the number of votaries to this fascinating form of exercise. A monthly handicap competition for a medal takes place amongst the members. As to tennis, many champion players have gone out of Cheltenham, both ladies and men, and many still remain here. The East Gloucestershire Tennis and Cricket Club possesses its own ground, and enrolled upon its list of members are the names of all the athletic *élite* of the town. There are several good riding masters, with large studs; there are also numerous livery stables, and the town is well supplied with hackney carriages, the drivers being of course all licensed, and the charges regulated by the Municipal Authority.

The country round about Cheltenham is very charming. Through lovely leafy lanes one may walk, ride, or drive into the surrounding villages. At the town of Tewkesbury, nine miles away, where the Warwickshire Avon joins the Severn, is the fine old Norman Abbey, and many quaint remnants of mediæval buildings are to be found in the town. Here it is possible to roam the broad meadows by Avonside, the site of the battle of Tewkesbury, or take a boat upon either river from amongst the large number of excellent craft that will be found on hire. Of the two, the Avon is the favourite stream for boating, and over the calm water in summer time it is sweet to glide through its verdant reaches up to Strensham Lock, and beyond to Pershore and Evesham, or even to Shakspere's Stratford, should time and inclination permit. The rivers also attract anglers, who have formed themselves into a club, and are granted cheap return tickets by the railway companies. To the ancient town of Gloucester, with its cathedral, it is but nine miles from Cheltenham, and there are trains to and fro frequently, both on the Midland and Great Western lines. The cathedral towns of Hereford and Worcester are also within easy distance by rail, and express trains carry their passengers into either Bristol or Birmingham within the hour. Beyond Gloucester, the Forest of Dean, with its unequalled Sylvan scenery, is immediately reached, and the most beautiful river in England—the Wye—is so near as to allow of a long day being spent upon it, starting from Ross, and passing Monmouth, Symonds' Yat, Tintern, to Chepstow, with time left to return to Cheltenham the same evening, if that be desired. But perhaps the most frequented place on the outskirts of Cheltenham in the summer time is Birdlip, which lies over Leckhampton Hill, six miles away. The views obtained upon the road are especially grand, and the extensive beech woods situated there, which are open to the public, afford an excellent place to picnic. Birdlip is on the road to the beautiful Stroud valley, the town of Stroud itself being thirteen miles distant from Cheltenham. Another favourite rendezvous for excursionists is Chedworth, situated in the Cotswold hills, and reached by rail, or after a pleasant drive of eleven miles. Here are some beautiful woods, but the interest centres in the remains of a Roman villa which were laid bare a few years ago. Large portions of mosaic pavement, Roman hot-air baths, &c., *in situ*, and other interesting relics, in a wonderfully good state of preservation, are here to be seen. Speaking of antiquities, the remains of ancient Roman entrenchments are of very common occurrence along the line of the Cotswold range and upon most of the outlying hills, the hills having been at one time evidently strongly held against a foe inhabiting the Severn valley or the land further to the westward. Several barrows also exist in the neighbourhood, and flint instruments are found in large numbers in the cultivated fields upon the hills.

It may be asked—How does all this affect the question of health? The answer is, that the success of a health-resort depends very largely upon the opportunities which the place affords for the amusement, exercise, and healthy recreation of the people who come to live in it. The class of invalids that repair to such a place as Cheltenham are not those who are stricken with acute forms of disease which must speedily end in death, or those hopeless chronic cases for whom a hospital for incurables would be the only appropriate provision. The people who come even for a short time are, with very few exceptions, enabled from the first to take part in whatever recreations are open to them, and upon such recreations and pleasing occupations their ultimate restoration to good health in great part depends; this is, in fact, part of their treatment, and not the least important part, though it is not desired to detract from the importance of following the advice of the medical practitioner as to a rigidly prescribed diet and mode of life, and the taking of baths, mineral waters, or any other remedial agent. To everyone conversant with general or special medical practice, a large number of cases will suggest themselves as being likely to derive benefit from a temporary or more permanent stay in Cheltenham, or such a place. In the practice of every physician are numerous cases of men and women who are found to be suffering from ailments engendered by the stress of their lives or the persistence in habits whose tendency is antagonistic to the welfare of the body, and the continued proper performance by the various organs of their functions. Let us take as an instance the conditions arising from irregularities of diet and the effect of alcoholic drink, taken in excess. Here there is congestion of the alimentary tract, which will be relieved by repeated daily doses of our natural medicinal waters and by exercise and change of habits. If not too far advanced, it is probable that every such case would benefit by a visit of a few weeks to Cheltenham, which is a desirable place for those in whom the gouty diathesis is apt to make itself apparent periodically in one of several ways, the attack being apparently less dependent upon evil habits than the cases mentioned above. Certain forms of dyspepsia, cases of anæmia and disordered nutrition, of obesity, of chronic constipation, of chronic lead or other metallic poisoning, of rheumatoid arthritis and other chronic rheumatic conditions, of locomotor ataxy and other affections having origin in the central nervous system, of hypochondriasis or mental debility, may all be benefited by a stay in Cheltenham under treatment by the methods here specially employed.

As a place of permanent residence, Cheltenham is, by reason of its mildness and salubrity, well suited for people of indifferent vitality. The town always has in it a great many elderly people, and the weekly death list is often surprising in the relatively large proportion of deaths of people at a very advanced age. This has on occasion been so remarkable as to lead to enquiries being made from the office of the Registrar General into the accuracy of the return. Of course the town has for long been a favourite place of settlement for retired army officers, and there are probably more "Old Indians" here than in any town in the country. The place appears to suit them very well, judging from the length of time they continue to live; and that the place and climate should suit the weakly as well as the old seems but a natural corollary. The beauty of the town proves attractive to many people who have been well treated at the hands of Fortune and are looking out for a place for private residence; a good many of this class are numbered in the population of the town.

But perhaps at the present time the influence which is strongest to determine families to settle here is found in the facilities afforded for the education of both boys and girls; the motto of the town, "*Salubritas et Eruditio*," being well chosen as representative of the two chief features in the interest of the place. In writing of Cheltenham as a health-resort, it would be out of place to enter into the advantages of Cheltenham as an educational centre, so that this subject must be dismissed with few words. The two interests in no way interfere with each other, but on the contrary are of mutual advantage. There is the Boys' College, which ranks as a great public school, and the Ladies' College, which as a high-class girls' school has perhaps no equal in the country, its classes ranging from the Kindergarten up to the advanced higher education which enables the final examinations for the London University degrees of Bachelor of Arts and Bachelor of Science to be passed by its determined students. Other important schools are the Dean Close Memorial School, the Cheltenham Grammar School, and the Training Colleges for school teachers, both men and women. All these of course help to keep up the population and status of the town.

There is a great plenitude of Society in Cheltenham. It is a friendly place, and whatever the class to which a person belongs he will here be able to make many acquaintances. There are numerous Clubs, political and other. The New Club, in the Imperial Square, numbers amongst its members most of the retired army and navy officers, with many others of the residents, and is non-political; but there are also political and other good Clubs. The balls and dinners, receptions and entertainments of other kinds, private and public, are endless. The Promenade presents a gay scene in the afternoon, when the broad pavements are thronged with well-dressed people. In the roadway, under the meeting boughs of the trees, roll the carriages, many of them splendidly horsed and equipped; whilst the band may be enlivening the scene with its music.

The vital statistics of Cheltenham show the town to have a low death-rate. It is considerably below the average for all England, and also below that for all Gloucestershire. For the ten years 1880—89 inclusive it averaged 17.3 per annum. Last year the death-rate in the three chief residential wards, which together have a population of upwards of 25,000, was 12.3 per thousand. The entire population of the recently-extended borough is 50,000 in round numbers. The average zymotic death-rate for the ten years was considerably less than one-half of the average rate for the whole country, being for the seven chief zymotics in most years less than 1 per thousand, and occasionally less than .5 per thousand. There appears to be no exceptional prevalence of any disease in Cheltenham of whatever sort, and the rate of incidence of certain diseases—*e.g.* diphtheria and whooping-cough—appears to be much below the average. The health statistics are likely to be still further improved in coming years, as all the new and approved appliances are being adopted by the Sanitary Authority. We have already a refuse destructor of the most effective kind, and extensive sewage farms, upon which the sewage is turned after deposition has been allowed to take place in tanks, the thicker matter being utilised along with the ashes from the destructor as a manurial compost, which is of such value as to be eagerly bought up by the numerous gardeners and growers of fruit who carry on a thriving industry in the suburbs. We have also a Washington Lyons' steam disinfector, and a public abattoir was opened last year and bids fair to be a great success. The Delancey Fever Hospital, a thoroughly well-built place, is about to be extended, to meet the increased accommodation required since the adoption of notification of infectious disease; and when the designs which have just been prepared have been carried into effect there will be a far larger provision for isolation of infectious diseases than is ordinarily met with in towns of this size. The town is well supplied with water, which is derived from springs situated in the Cotswold hills. The oolitic limestone here yields a water very free from organic impurity, and of quite a moderate degree of hardness; a better drinking water, in fact, could not be desired.

The following notes of the Meteorology of Cheltenham are taken from those supplied at the end of last year by Mr. Richard Tyrer, B.A., F.R. Met. S., of Cheltenham Modern School. Of the climate generally it may be said that it is mild. The maximum temperatures of the hottest months are lower by a few degrees than those of London and many towns of smaller size, and similarly the minimum temperatures of winter are higher. The humidity of the atmosphere averages a higher degree than that noticed in the East of England, but the rainfall is low, and the average number of days upon which any rain falls is moderate for this side of the country. The prevailing winds are marked in that segment of the compass circle included between N.W. and S., the general direction of the wind during the year 1892 being as follows:

| | | | |
|---|---|---|---|
| From the | North | ... ... ... | on 30 days. |
| " " | North-East | ... ... | " 17 " |
| " " | East | ... ... ... | " 15 " |
| " " | South-East | ... ... | " 27 " |
| " " | South | ... ... ... | " 53 " |
| " " | South-West | ... ... | " 77 " |
| " " | West | ... ... ... | " 61 " |
| " " | North-West | ... ... | " 28 " |

There were calms on 58 days.

The following table shows the average annual mean readings for the last 15 years:

| Atmospheric Pressure. | Air Temperatures. | | | | Humidity. | | Rainfall. | |
|---|---|---|---|---|---|---|---|---|
| | 9 a.m. | 9 p.m. | Max. | Min. | 9 a.m. | 9 p.m. | Inches. | Days. |
| 29.93 | 47.9 | 46.5 | 55 | 40 | 83 | 87 | 28.4 | 188 |

I am obliged for the present to postpone the precise description of the mineral waters of Cheltenham, as well as of the new buildings that it is proposed to immediately erect for their use, most extensive plans for which, including a magnificent system of baths, are at the present time under the consideration of the Committee; but in a later issue I trust to be able to add a short note upon the details of these subjects.

